# Clinically relevant inflammatory breast cancer patient-derived xenograft–derived *ex vivo* model for evaluation of tumor-specific therapies

**DOI:** 10.1371/journal.pone.0195932

**Published:** 2018-05-16

**Authors:** Bedrich L. Eckhardt, Maria Gagliardi, LaKesla Iles, Kurt Evans, Cristina Ivan, Xiuping Liu, Chang-Gong Liu, Glauco Souza, Arvind Rao, Funda Meric-Bernstam, Naoto T. Ueno, Geoffrey A. Bartholomeusz

**Affiliations:** 1 Department of Breast Medical Oncology, The University of Texas, MD, Anderson Cancer Center, Houston, Texas, United States of America; 2 Morgan Welch Inflammatory Breast Cancer Research Program and Clinic, The University of Texas, MD, Anderson Cancer Center, Houston, Texas, United States of America; 3 Department of Experimental Therapeutics, The University of Texas, MD, Anderson Cancer Center, Houston, Texas, United States of America; 4 Department of Investigational Cancer Therapeutics, The University of Texas, MD, Anderson Cancer Center, Houston, Texas, United States of America; 5 Nano3D Biosciences, Houston, Texas, United States of America; 6 University of Texas Health Science Center, Houston, Texas, United States of America; 7 Department of Bioinformatics and Computational Biology, The University of Texas, MD, Anderson Cancer Center, Houston, Texas, United States of America; University of South Alabama Mitchell Cancer Institute, UNITED STATES

## Abstract

Inflammatory breast cancer (IBC) is a rare and aggressive presentation of invasive breast cancer with a 62% to 68% 5-year survival rate. It is the most lethal form of breast cancer, and early recognition and treatment is important for patient survival. Like non-inflammatory breast cancer, IBC comprises multiple subtypes, with the triple-negative subtype being overrepresented. Although the current multimodality treatment regime of anthracycline- and taxane-based neoadjuvant therapy, surgery, and radiotherapy has improved the outcome of patients with triple-negative IBC, overall survival continues to be worse than in patients with non-inflammatory locally advanced breast cancer. Translation of new therapies into the clinics to successfully treat IBC has been poor, in part because of the lack of *in vitro* preclinical models that can accurately predict the response of the original tumor to therapy. We report the generation of a preclinical IBC patient-derived xenograft (PDX)-derived *ex vivo* (PDXEx) model and show that it closely replicates the tissue architecture of the original PDX tumor harvested from mice. The gene expression profile of our IBC PDXEx model had a high degree of correlation to that of the original tumor. This suggests that the process of generating the PDXEx model did not significantly alter the molecular signature of the original tumor. We demonstrate a high degree of similarity in drug response profile between a PDX mouse model and our PDXEx model generated from the same original PDX tumor tissue and treated with the same panel of drugs, indicating that our PDXEx model had high predictive value in identifying effective tumor-specific therapies. Finally, we used our PDXEx model as a platform for a robotic-based high-throughput drug screen of a 386-drug anti-cancer compound library. The top candidates identified from this drug screen all demonstrated greater therapeutic efficacy than the standard-of-care drugs used in the clinic to treat triple-negative IBC, doxorubicin and paclitaxel. Our PDXEx model is simple, and we are confident that it can be incorporated into a PDX mouse system for use as a first-pass screening platform. This will permit the identification of effective tumor-specific therapies with high predictive value in a resource-, time-, and cost-efficient manner.

## Introduction

Inflammatory breast cancer (IBC) is a rare, clinically and pathologically unique breast cancer subtype [1] that accounts for 2% to 6% of all breast cancers diagnosed in the United States [2–6] but 7% to 10% of all breast-cancer-related deaths [6–10]. It is the most aggressive subtype of breast cancer and associated with a poor prognosis [6,11–13], due in part to the high frequency of misdiagnosis at the onset of IBC [14–17] and its propensity to rapidly metastasize [18]. Some patients with IBC present without an underlying palpable mass [17,19–20]. The 5-year overall survival rate for patients with primary IBC is typically 62% to 68% [2, 21]. In an attempt to improve this unfavorable outcome, the International Expert Panel on IBC proposed clinical guidelines important for the diagnosis of this disease [17,19, 22]. These guidelines state that in order for IBC to be diagnosed, the clinical presentation should include erythema, edema, peau d’orange, and/or warm breast with or without an underlying palpable mass and a duration of no more than 6 months with erythema occupying at least one-third of the breast. Although patients with primary IBC present with characteristic clinical signs and symptoms [2], the molecular mechanisms modulating these presentations are poorly understood.

Van Laere et al [23] showed that IBC is a heterogeneous disease comprising luminal, HER2-positive, and triple-negative subtypes. The triple-negative subtype of IBC is overrepresented [21, 23], accounting for 20% to 40% of all IBC cases. Although improvements in IBC patient survival have been noted with the introduction of trastuzumab-based systemic therapy to treat patients with the HER2-expressing subtype [21, 24–29], this improved outcome is not seen in the triple-negative IBC group, who are excluded from hormonal therapy and HER2 targeting as treatment options [30–31]. In the absence of any known druggable target, treatment of patients with triple-negative IBC follows the standard-of-care treatment prescribed to patients with triple-negative non-inflammatory breast cancer [1], which is a combination of taxane- and anthracycline-based neoadjuvant chemotherapy followed by modified radical mastectomy including axillary clearance and postoperative chest wall and/or nodal radiotherapy [1, 14, 24]. The reasons for the poor outcomes of treatment in patients with triple-negative IBC are the limited treatment options [23] and onset of resistance to the standard of care therapy [5, 21, 32–34]. There is thus an urgent need to identify new and effective therapies to prolong the disease-free survival of patients with triple-negative IBC.

One of the reasons for the lack of success to date in identifying effective therapies for triple-negative IBC is the use of simplified *in vitro* cell culture models that fail to recapitulate the complex 3-dimensional (3D) tumor microenvironment as screening platforms to identify new drugs [35–37]. Another reason for the lack of success to date is that the cell lines used in these models may be genetically distinct from tumor cells in patients as a result of their adaptation to growth outside a natural tumor microenvironment [38]. Although complex 3D multicellular tumor models that recapitulate important aspects of the 3D microenvironment have shown promise in the development of new anticancer therapies [35, 37, 39–45], these 3D models fail to replicate tumor heterogeneity [46]. Currently, the most effective preclinical models are patient-derived xenograft (PDX) models [38]. PDX models recapitulate the heterogeneity of tumors in patients and demonstrate genetic stability that closely and stably replicates the human tumor microenvironment [38, 47]. PDX models respond to the standard-of-care therapies in a manner similar to that observed in patients and have played a major part in furthering our understanding of cancer and our ability to develop new effective therapies against cancer. The generation of PDX models, however, requires immense resources and is costly and time-consuming, and thus PDX models have limited utility as platforms for high-throughput drug screens [38, 48]. *Ex vivo* culture models [39] and organoid models [49–50] are cheaper alternatives that are currently gaining popularity as clinically relevant preclinical models for the development of new and effective anticancer drugs. As these models recapitulate most of the characteristics of the original tumor, they are useful for studying tumor biology and developing tumor-specific therapies.

Herein, we chronicle the development of an IBC PDX-derived *ex vivo* (PDXEx) model generated from the cellular milieu released from a PDX tumor harvested from a mouse. Our PDXEx model closely replicates the tissue architecture of the original PDX tumor. We show a highly significant correlation between the gene expression signatures of the *ex vivo* tumor tissue model and the original PDX tumor, suggesting that our PDXEx model retains certain complex intra- and extra-cellular communication networks, cell signaling pathways, and differentiated cell types characteristic of the *in vivo* tumor and thus will have physiological responses similar to those observed in the *in vivo* model. We confirm the value of our model in terms of predicting effective tumor-specific therapies and demonstrate its potential application as a drug screening platform to identify novel and effective IBC tumor-specific therapies. Generating the PDXEx model was time, resource, and cost effective, and we predict that incorporating this PDXEx model as a first-pass screening platform within a PDX system will permit the cost- and time-efficient identification of therapies with a high probability of efficacy against the original tumor. Combining the PDXEx model with a PDX system has the potential to identify new therapies that would result in benefit if incorporated into an individual patient’s adjuvant therapy regimen.

## Materials and methods

### Culture medium

Advanced DMEM (Gibco Life Technologies) was supplemented with Pen/Strep (50 units/ml), antibiotic/antimycotic (1x), glutamine (4 mM), epidermal growth factor (20 ng/ml), and 0.5% fetal calf serum.

### Generation of tumors in mice

Animal experiments were conducted in full compliance with MDACC Institutional Animal Care and Utilization Committee (IACUC) policies and procedures (protocol ID: 00001305-RN00). Mice were housed under pathogen-free conditions and treated in accordance with NIH guidelines and standard rodent chow and water were available ad libitum. Tumors were implanted into NSG mice under Ketamine/Xylazine light anesthesia and Ketoprofen was used as an analgesic. At time of tumor harvest, mice were euthanized through CO2 inhalation followed by secondary exsanguination Previously archived, frozen IBC PDX tissue fragments (~100 mg) of the Bcx087 IBC PDX model were thawed and rinsed in PBS. Pieces of tumor tissue were mechanically fragmented with scissors prior to digestion in 3 mL of collagenase A (1 mg/mL solution in PBS) for 15 minutes at 37°C. Digested tissue samples were filtered through a 70-μm pore strainer, and the collagenase A was inactivated by addition of an equal amount of DMEM supplemented with 10% fetal calf serum. Samples were pelleted by centrifugation (400 x g, 2 minutes, room temperature) and resuspended in 1 mL of red cell lysis buffer (Roche) for 2 minutes. Samples were centrifuged, and the cell pellet was resuspended in a 50:50 PBS:Matrigel solution to a final concentration of 2.0x10^7^ cells/mL. Next, 100 μL of the PDX cell suspension was implanted into the mammary fat pad of 6- to 8-week old nude mice (*Nu/Nu* mice) following the protocol approved by the Institutional Animal Care and Use Committee at The University of Texas MD Anderson Cancer Center. Tumor growth in the mice was monitored once per week until tumors reached a volume of 500 mm^3^ to 800 mm^3^. Mice were monitored for signs of morbidity: failure to reach food and water; failure to thrive; inappropriate behavior such as lack of grooming, circling, etc.; a hunched posture, lateral recumbency, or distended abdomen; dyspnea; or weight loss >20%. Moribund animals were euthanized, and tumors and other tissues were removed for weighing and molecular marker studies.

### Harvest of tumors

Prior to tumor harvest, mice were euthanized in a chamber containing CO2 from a cylinder as recommended by the American Veterinary Panel on Euthanasia [51]. Every effort was made to minimize the pain and discomfort of all animals used in the proposed work.

### Generation of PDXEx models

**Fig 1:**
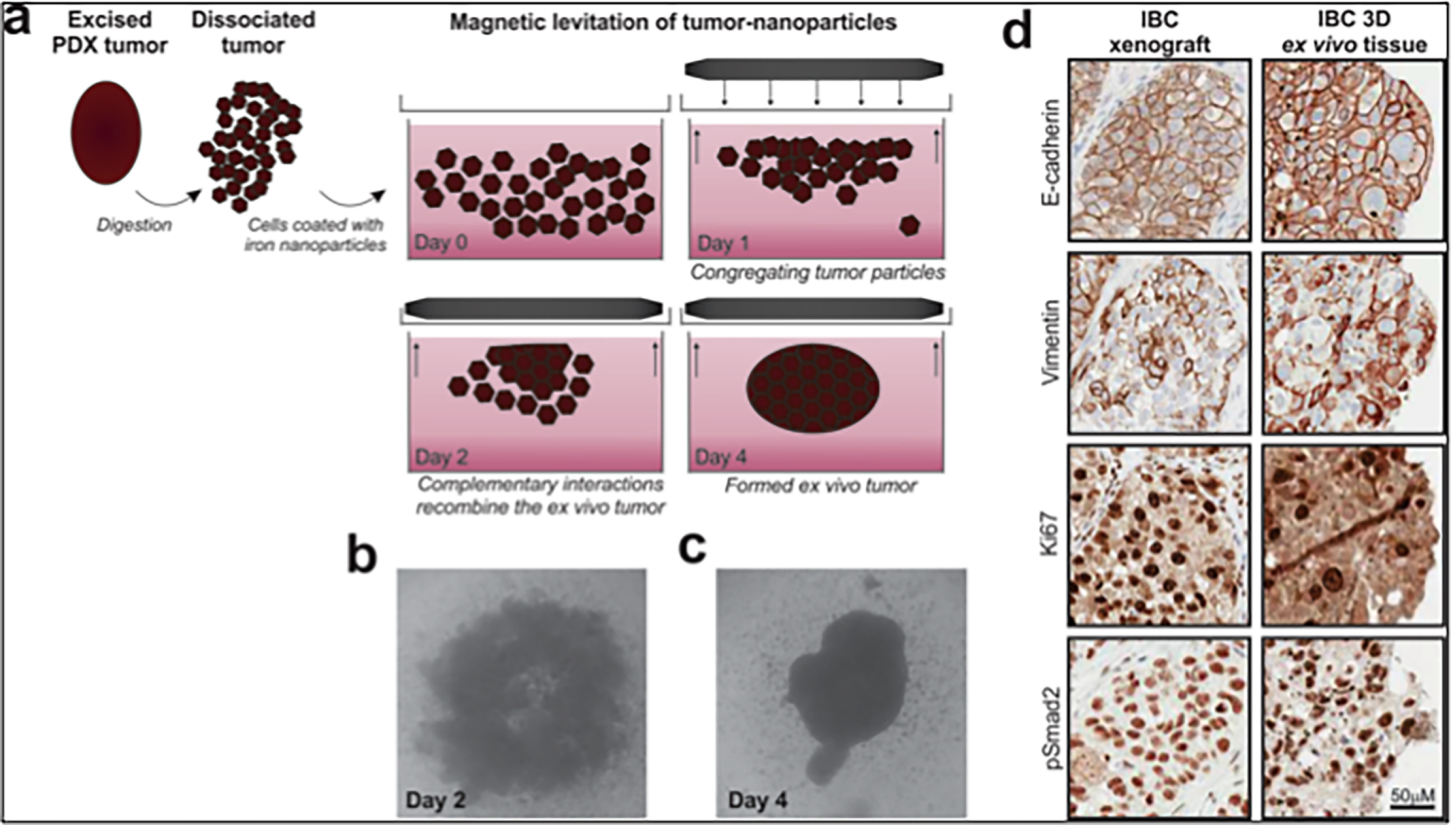
Generation of preclinical PDX-derived ex-vivo model. (A) A freshly harvested PDX tumor from a mouse was finely chopped to release all its cellular content. The released cells are filtered to separated them form fat and necrotic tissue and tagged with a nanoparticle assembly of iron oxide and iron nanoparticles cross-linked with poly-L-lysine (NanoshuttleTM) by an overnight incubation prior to been placed under a magnetic field. (Bio-AssemblerTM) (n3D Biosciences Inc.). (B) The levitating mass of cells developed into a loose unstructured mass by day 2 of incubation and (C) into a more structured compact mass by day 4 of incubation. (D) Immunohistochemistry analysis of PDX tissue and PDXEx tissue revealed a similar tissue architecture and staining for E-cadherin, Vimentin, Ki67 and pSMAD2.

Freshly harvested PDX tumors were introduced into 10 mL of room-temperature PBS in a sterile 50-mL centrifuge tube and delivered to the laboratory within 30 minutes. Tumors were photographed, weighed, and placed in a 35-mm sterile tissue culture dish containing culture medium. Tumors were mechanically chopped using a pair of sterile scalpels to release the cellular milieu. The cell suspension was passed through a 100-μM filter to separate the homogeneous cell suspension from tissue aggregates, fat, and necrotic tissue, and the concentration of the cell suspension was determined. *Ex vivo* tumor tissue was generated by magnetic levitation using the Bio-Assembler^TM^ (Nan03D Biosciences,Inc.) as previously described [52]. Briefly, cells were incubated with nanoshuttle^TM^ (nanoparticle assembly of iron oxide and iron nanoparticles cross-linked with poly-L-lysine) for about 18 to 24 h at a ratio of 1 μL of nanoshuttle^TM^ to 150,000 cells to permit the binding of the iron nanoparticles to the surface of the cells. The nanoshuttle-coated cells were then placed in the Bio-Assembler system to bring the cells into a levitating aggregate (Fig 1A). Cells were maintained within the Bio-Assembler system for 10 to 14 days at 37°C in a humidified environment with medium changed every 3 to 4 days. The best results were obtained from cells released from PDX tumors that were vascular with a fat content between 20% and 30% of the total tumor weight. Tumors weighing 0.8 gram to 1 gram generally yielded the best results.

### Magnetic bio-printing of PDXEx to generate an *in vitro* screening platform

**Fig 2:**
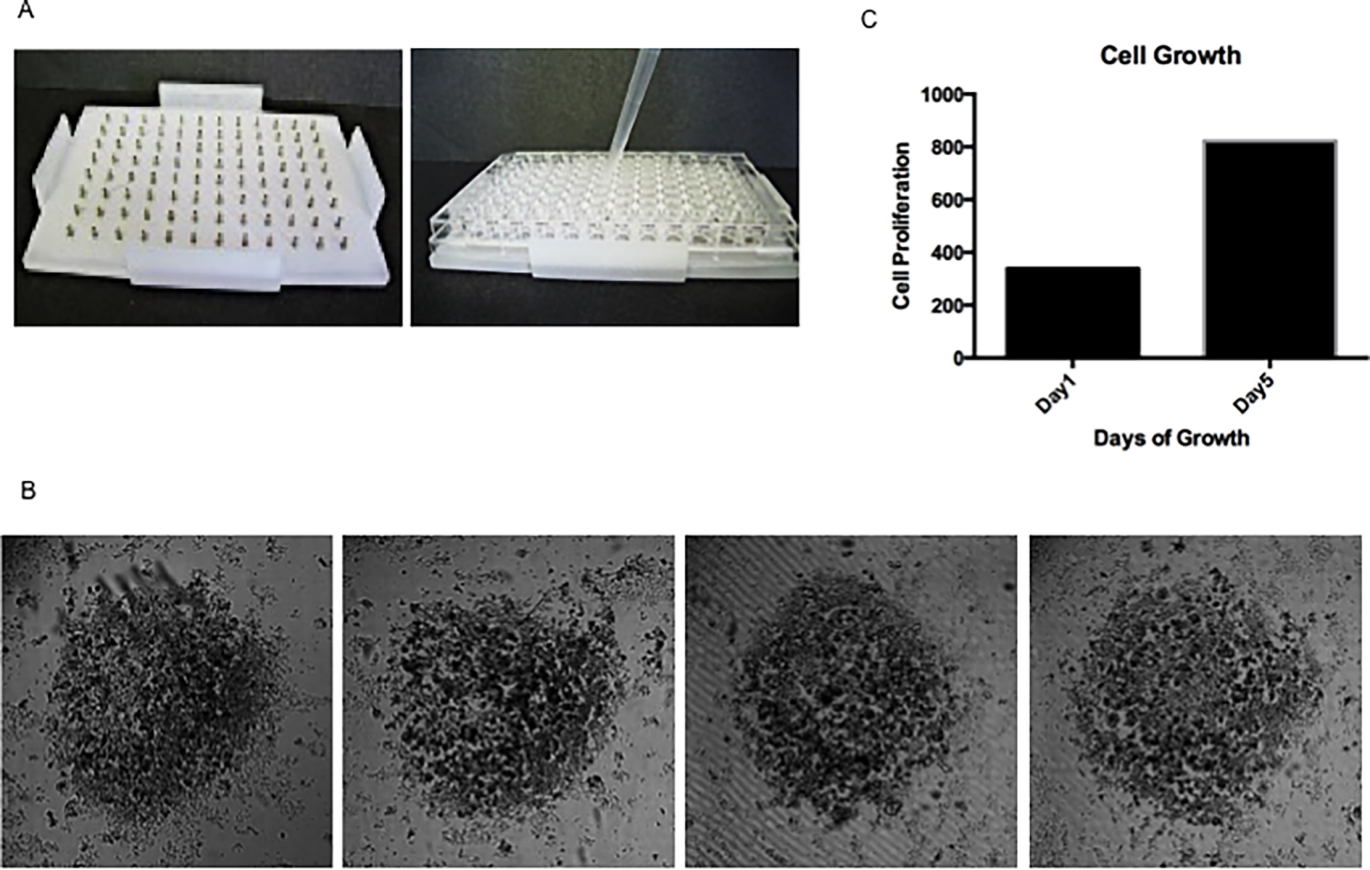
Tissue characteristics of the ex-vivo tumor model. (A) Bio-printing cells–Tumor cells tagged with a nanoparticle assembly of iron oxide and iron nanoparticles cross-linked with poly-L-lysine (NanoshuttleTM) were dispensed into an ultralow attachment 96 well tissue culture plate placed on a 96 well magnetic drive (n3D Biosciences, Inc). (B) Morphology (4x magnification) of the ex-vivo tumor tissue prints after 5 days of growth at 37°C revealing uniformly sized structures. (C) Proliferative capacity of the PDXEx tissue following 5 days of culture at 37°C.

Cells harvested from IBC PDX tumors were incubated overnight with nanoshuttle^TM^ as described in the preceding section. The iron nanoparticle–coated cells were then dispensed into a 96-well tissue culture plate placed on a 96 well magnetic drive (Fig 2A) at a final concentration of 75,000 cells per well and incubated at 37°C in a humidified environment.

### Purification of total RNA

Total RNA isolated from the PDX tumors as well as from the *ex vivo* tumor tissue after 5 days of growth in culture was purified with the Trizol method (Invitrogen) according to the manufacturer’s instructions.

### Whole transcriptome expression profiling

Whole transcriptome expression profiling was performed using an Affymetrix GeneChip human Clariom D array (PN 902923, Life Technologies). Total RNA sample labeling and processing, GeneChip hybridization, and scanning were performed according to instructions in the Affymetrix user manual of GeneChip WT PLUS Reagent Kit–“Target Preparation for GeneChip Whole Transcript Expression Arrays (P/N 703174)”. Briefly, the first-strand cDNA was reverse synthesized from 100 ng of total RNA spiked in controls using a whole transcript plus kit, using primers containing a T7 promoter sequences (Genset, La Jolla, CA). cRNAs were generated and amplified by *in vitro* transcription from double-strand cDNA templates containing T7 promoter. Fifteen micrograms of purified cRNA was reverse transcribed into second-cycle single-strand sense cDNA using second-cycle primers and dNTP mix with fixed dUTP/dTTP ratio in reaction mix. The single sense strand cDNA in second cycle was released from RNA/DNA hybrids by RNase H digestion. Then 5.5 μg of purified sense-strand cDNA was fragmented by uracil-DNA glycosylase and apurinic/apyrimidinic endonuclease 1 at the unnatural dUTP residues to break the DNA strand. The fragmented cDNA was labeled by terminal deoxynucleotidyl transferase using the Affymetrix proprietary DNA labeling reagent, which is covalently linked to biotin. Five micrograms of biotin-labeled sense-strand cDNA fragments in 200 μL of hybridization mix was loaded onto a human Clariom D array and hybridized to probes on the array in a GeneChip Hybridization Oven 645 (Affymetrix) for 16 h at 45°C with 60 rpm rotation. After hybridization, each array was washed and stained with streptavidin–phycoerythrin (Invitrogen), and the signal was further amplified with biotinylated anti-streptavidin antibody (Vector Laboratories) on the GeneChip Fluidics Station 450 (Affymetrix). Arrays were scanned with the GeneArray G7 scanner (Affymetrix) and quantified into CEL files with image and signal intensities.

### Bioinformatic analysis

The CEL files generated were analyzed through Transcriptome Analysis Console 4.0, which normalizes (and applies the log2 function to) array signals using a robust multiarray averaging algorithm. The Spearman's rank-order correlation test was applied to measure the strength of the association between gene expression in different arrays. The graphical representation of data and statistical analysis were performed in R (version 3.0.1) (http:///www.r-project.org/), and statistical significance was defined as p less than 0.05.

### Immunohistochemistry

Excised tumor tissue was fixed in formalin and archived in paraffin. For analysis of proteins by immunohistochemistry, 5-μm slides were cut from archived paraffin blocks and subsequently adhered to glass slides. The sections were deparaffinized, rehydrated, and subjected to citrate buffer antigen retrieval. Staining was performed on an automated immunohistochemical autostainer (Lab Vision Corp.). Briefly, the slides were blocked for nonspecific binding and incubated with relevant antibodies for 1 hour at room temperature. The primary antibody was detected with the labeled streptavidin biotin plus peroxidase kit (DAKO). Slides were developed with DAB chromogen to reveal immunoreactivity, counterstained with hematoxylin, and covered with a glass coverslip. Antibodies used for immunohistochemistry were as follows: anti-E-Cadherin rabbit mAb (1:200) (Cell Signaling Technology, clone 24E10, #3195), anti-Vimentin XP rabbit mAb (1:200) (Cell Signaling Technology, clone D21H3, #5741), anti-Ki67 rabbit mAb (1:200) (GeneTex, GTX16667), and anti-pSmad2 (Ser465/467) rabbit mAb (1:200) (EMD Millipore, clone A5S, #04–953).

### Statistical analysis of the high-throughput anticancer drug screen

Median scores of viability across 3 replicates were normalized relative to the plate-level controls (i.e., the median viability was divided by the median viability of the controls). The resulting values were then ranked from lowest to highest. Drugs ranking highest exhibited the strongest reduction in viability relative to the control. The statistical analysis for the in-vivo data described in the section “Value of the PDXEx model for predicting tumor response to drugs” is present in the paper by KURT et al. [53].

### High-throughput anticancer drug screen

Using robotics, 10 μL of the small molecules comprising the Anti-Cancer 386 Compound Library (Selleckchem) were dispensed into each well of a 96-well bio-printed PDXEx. Each well in the array contained 100 μL of medium. The small molecules were dispensed into 3 sets of plates for final concentrations of 1 μM, 0.5 μM, and 0.25 μM. Each drug concentration set was performed in triplicate. The bio-printed PDXEx was incubated with the drug concentrations for 5 days. Viability of the *ex vivo* tumor tissue was determined on day 5 using the Cell Titer Glo Luminescent cell viability assay (Promega).

## Results and discussion

### Generation of the PDXEx model

Using previously described methodology [52], the cellular milieu released from a Bcx087 (triple-negative IBC) PDX tumor was tagged with a nanoparticle assembly of iron oxide and iron nanoparticles cross-linked with poly-L-lysine (Nanoshuttle^TM^) and placed under a magnetic field (Bio-Assembler^TM^), resulting in the cells’ been drawn together into a levitating mass (Fig 1A). Prolonged incubation of the levitating cells at 37°C resulted in a morphological change of this cellular mass. A loose, poorly organized structure was observed after 2 days of incubation (Fig 1B), and this structure gradually transformed into a more structured compact mass by day 4 (Fig 1C). To determine the similarity between the tissue architecture of the original PDX tumor and the PDXEx model, tissue sections were generated from the respective samples and analyzed by immunohistochemistry. We observed a high degree of similarity between the tissue architecture of the 2 sets of tissue (Fig 1D). In addition, subpopulations of IBC cells within tumor xenografts and our PDXEx tissues were found to be Ki67 positive, which is indicative of active proliferation in both tissues. Positive staining was also for E-cadherin, Vimentin, and pSMAD (Fig 1D). E-cadherin is highly expressed in IBC [54]. The similarities between the 2 sets of tissue samples with respect to tissue architecture and expression levels of these biomarkers suggested that the PDXEx model closely recapitulates the tumor microenvironment of the original PDX tumor.

### Comparison of molecular signatures of the original IBC PDX tumor and the IBC PDXEx model

Having demonstrated that our IBC PDXEx model retained both the tissue architecture and expression profile of key biomarkers of the original PDX tumor, it was important to confirm that our model also retained the molecular signature of the original PDX tumor, which would allow our model to serve as a relevant preclinical platform for high-throughput drug screens. To compare the molecular signatures, we utilized magnetic 3D bio-printing (n3D Biosciences^TM^, Inc.), which has been shown to have a wide range of applications in tissue engineering [55–60]. Briefly, viable cells released from the PDX tumor and incubated with poly-L-lysine–tagged magnetic nanoparticle assembly for 24hr were harvested and dispensed into a 96-well ultra-low attachment tissue culture plate at a concentration of 50,000 cells per well, and the plate was placed on a bed of magnets (Fig 2A). Well-formed and equally sized PDXEx “bio-prints” were observed at 5 days of incubation, a time corresponding to the duration of the planned high-throughput drug screens (Fig 2B).

We predict that using the cellular milieu of an entire tumor to generate the PDXEx bio-prints for use as a screening platform will results in the majority of the cellular components of the heterogeneous tumor being distributed throughout the PDXEx bio-print. At present, we typically harvest 60 million to 90 million cells from a single PDX tumor weighing approximately 0.8 to 1.2 grams. This yield of cells permits the bio-printing of approximately twelve 96-well-plate arrays. This number is sufficient to perform a high-throughput drug screen in triplicate utilizing a 380-compound drug library. We are currently working towards developing a 384-well tumor tissue array to screen larger drug libraries.

For our preclinical PDXEx model to serve as a clinically relevant screening platform, (i) the cells in the model must retain the capacity to proliferate in culture to enable relatively rapid assessment of the anti-proliferative activity of novel therapeutic agents, and (ii) the method used to produce the model must produce robust and reproducible results with respect to similarity between the genetic signature of the original PDX tumor and the genetic signature of the PDXEx model. To assess the proliferative capacity of our PDXEx model, the viability of the bio-printed tissue was determined at 24 hours and 5 days after printing using the Cell Titer Glo Luminescent cell viability assay. The rationale for determining cell viability after 5 days of culture is that when the bio-printed PDXEx platform is used for drug screens, the bio-printed PDXEx will be grown in the presence of the drugs for 5 days before viability is measured. Our data clearly showed tissue proliferation during this 5-day period (Fig 2C).

**Fig 3:**
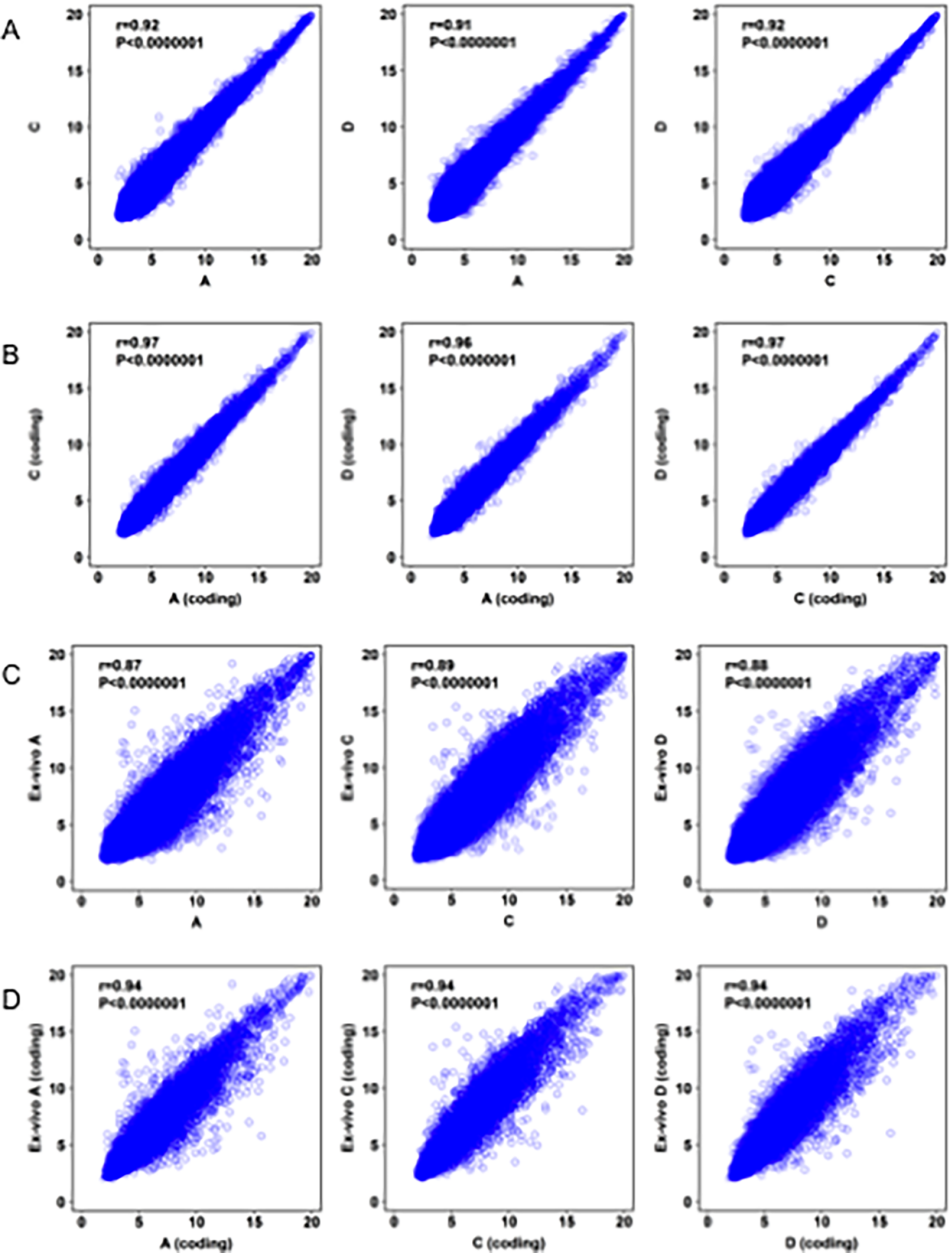
Correlation of gene expression profiles between three PDX tumors harvested from mice implanted with tissue from the same original PDX tumor. (A) Highly significant correlation between the entire gene set between tumor A and C, A and D and D and C (B) Selecting the coding genes in this analysis increased the significance of correlation. (C) Highly significant correlation between the entire gene set between each PDX tumor and its corresponding PDXEx tumor model (D). Selecting the coding genes in this analysis increased the significance of correlation.

To evaluate the robustness and reproducibility of the method used to generate the PDXEx model, we performed the method 3 times using PDX tumors harvested from 3 host mice, all of which had been implanted with the same original PDX tumor tissue. To ensure that the harvested PDX tumors had similar genetic signatures, we isolated purified RNA from these tumors and analyzed it using Clarion D human Affymetrix arrays comprising more than 6,765,500 probes derived from >134,700 characterized genes of both poly(A) and non-poly(A) mRNA, to provide complete and unbiased coverage of the transcriptome and profile differentially expressed genes. This complete gene set comprises coding genes, noncoding genes, microRNA, precursor micro RNA, pseudogenes, small RNA, and unassigned genes. The Spearman rank correlation was used to test the strength of the association in gene expression profiles between the 3 PDX tumors. We observed a highly significant genetic correlation (>90%) between the PDX tumors harvested from the 3 mice (Fig 3A). A higher degree of correlation (>96%) was observed when only the coding genes were selected (Fig 3B). These data indicated that the host mice exerted a minimal effect on the genetic signature of the original PDX tumor.

Next, in a similar manner, we determined the significance of the association in gene expression profiles between each PDX tumor and the resulting PDXEx model. Here too, a highly significant correlation (>87%) was observed between the gene expression profiles within each set (Fig 3C), and a higher correlation (>90%) was observed when only the coding genes were selected (Fig 3D).

**Fig 4:**
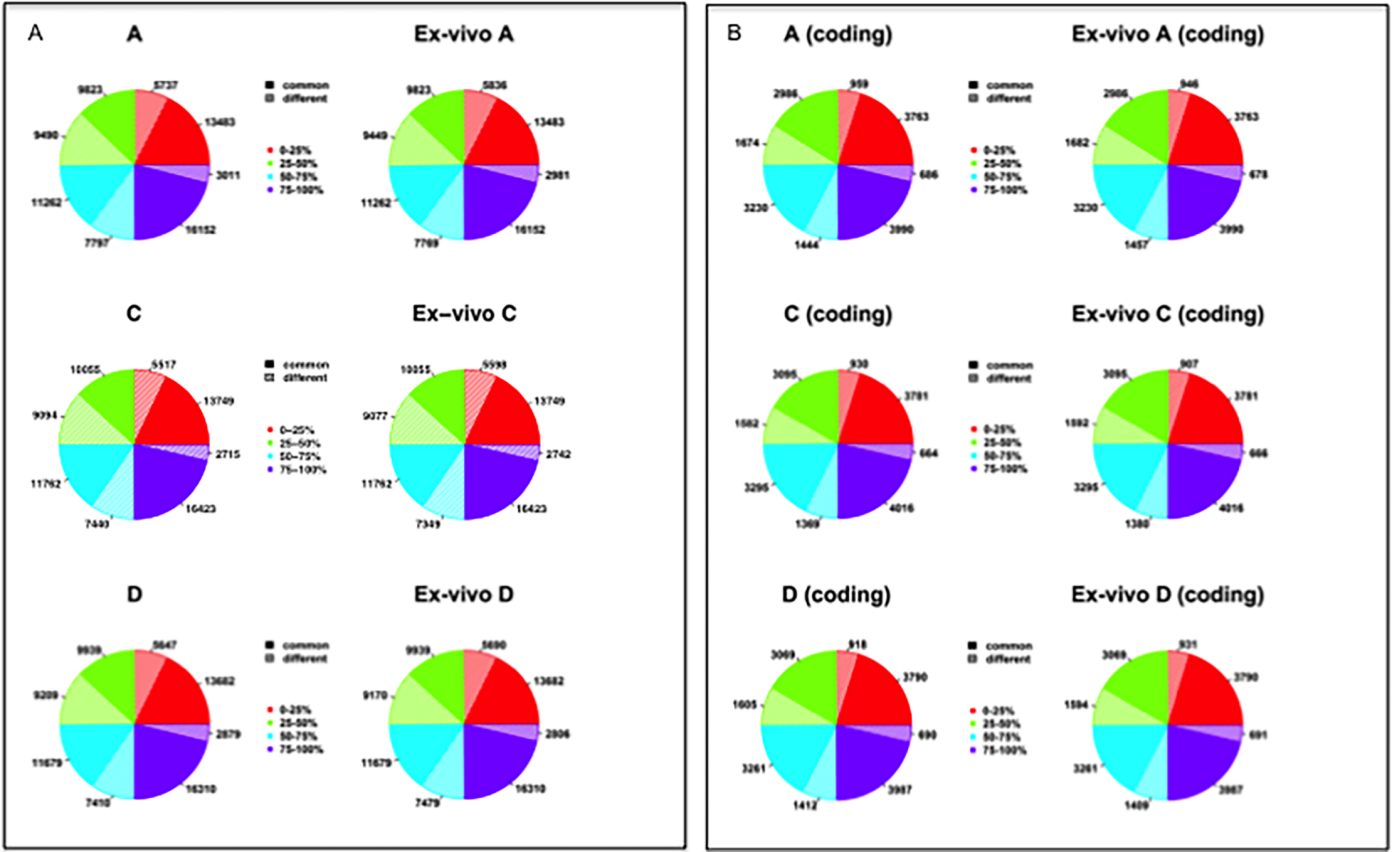
Comparison of genes either similarly or differentially expressed. (A) Entire gene set divided into 4 quantiles based on their level of expression to enable determination percentage of similarly expressed genes and differentially expressed genes. (B) Coding gene set divided into 4 quantiles based on their level of expression to enable determination percentage of similarly expressed genes and differentially expressed genes.

We next determined the proportions of genes that were similarly expressed and differentially expressed between each PDX tumor and the resulting PDXEx model. We first performed the analysis with the entire gene set (Fig 4A) and then performed the analysis with only the coding genes (Fig 4B). For the sake of completeness, the genes were divided into 4 quantiles according to their expression levels. For original PDX tumor A, patterns were as follows: for genes in the top quantile (76%-100%), 59% of all genes and 69% of coding genes had similar expression levels between the PDX tumor and the resulting PDXEx model; for genes in the third quantile (56%-75%), 84% of all genes and 85% of coding genes had similar expression levels; for genes in the second quantile (26%-50%), 51% of all genes and 64% of coding genes had similar expression levels; and for genes in the first quantile (0–25%), 70% of all genes and 80% of coding genes had similar expression levels. Comparisons of gene expression between the original PDX tumor and PDXEx model for original PDX tumors B and C showed similar results (Fig 4A and 4B). We speculate that the differences between quadrants in the ratios of similar and differential gene expression observed in each PDX/PDXEx tumor model set are associated with the heterogeneity of each tumor and PDXEx model and not a result of a genetic drift resulting from the generation of the PDXEx model nor the biocompatibility of the nanoparticles. Studies previously performed to address the biocompatibility of the nanoparticles [52, 57, 60–66] confirmed that the nanoparticles are bio-inert as they do not affect cell viability, oxidative stress, protein expression, inflammatory response, or stem cell differentiation. Our finding that the majority of genes and especially the majority of coding genes had similar expression in PDX tumors and their PDXEx models suggests that our model recapitulates important properties of the tumor microenvironment of the original tumor. This possibility needs further investigation, which is currently under way.

**Fig 5:**
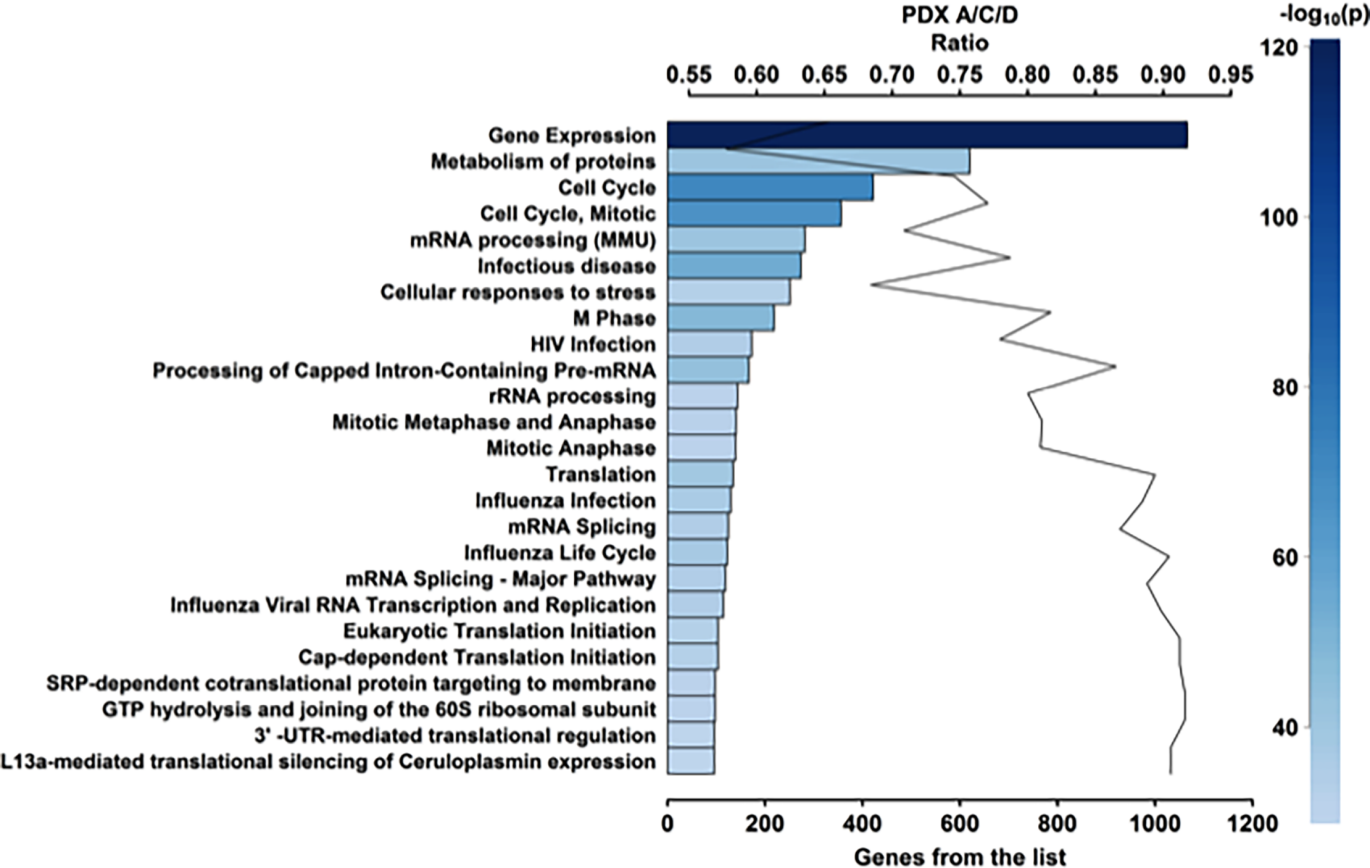
Layout of biological pathways common to all 3 PDX tumors. The Top 25 out of 1116 pathways are presented.

The lack of clearly defined molecular drivers for IBC contributes to the difficulty of identifying effective therapies to treat this disease. We too were unable to identify unique molecular drivers of the Bcx087 IBC PDXEx model. In Table 1, we present the top 50 genes based on their expression levels. Twenty-two of these genes are members of the family of ribosomal proteins. Two potential targets within this group are the redox regulator thioredoxin-1 (*Txn-1*) and aspartate beta-hydroxylase (*ASPH*). Thioredoxin-1 [67–69] and aspartate beta-hydroxylase [70] are highly expressed in many cancers and contribute to the aggressive nature of these cancers. We are currently investigating the role of these molecules in the biology of IBC and determining if targeting these molecules will improve our ability to treat this very aggressive subtype of breast cancer. Applying gene ontology to our derived expression data, we identified several distinct biological pathways that are associated with Bcx087 tumors (Fig 5) (Table 2). As IBC is a rapidly growing tumor, it is not surprising that the pathways with the highest numbers of highly expressed genes included those regulating the cell cycle, metabolism, and protein metabolism.

**Table 1:** Gene expression levels of the top 50 ranked genes.

Gene Symbol	Fold Expression	Gene Symbol	Fold Expression
**RPS7**	19.928	PPIA	18.838
**RPL30**	19.928	RPS6	18.837
**TXN**	19.928	HSPE1	18.81
**RPS21**	19.725	MTRNR2L8	18.8
**RPD20**	19.723	CANX	18.763
**ASPH**	19.578	RPL38	18.762
**LAPTM4A**	19.545	CRABP2	18.637
**RPL41**	19.51	TCEB1	18.617
**RPS15A**	19.508	SNRPE	18.613
**HSP90AB1**	19.502	RPS24	18.59
**RPL35A**	19.453	RPL9	18.553
**HSPA8**	19.445	YWHAQ	18.515
**RPS23**	19.438	SOD1	18.29
**RPL37A**	19.323	RPN1	18.242
**THEM123**	19.272	RPL39	18.215
**COX6C**	19.2	RPL31	18.155
**TMSB10**	19.168	RPL7	18.145
**COX7C**	19.167	NDUFA1	18.122
**NDUFS5**	19.065	VKORC1	18.115
**H3F3A**	19.054	RPS12	18.102
**RPS8**	19.028	TPT1	10.062
**ITGB1**	18.97	RPL27	18.023
**EEF1A1**	18.917	ATP6VOE1	18.081
**UBB**	18.89	ILF2	18.015
**RPL34**	8.882	RPL5	17.94

**Table 2:** Top 25 pathways among 1116 identified to be activated in the Bcx087 IBC model.

Pathway	Number	Total	pValue
**Gene expression**	1066	1631	3.10E-121
**Cell Cycle**	422	566	3.40E-72
**Cell Cycle, Mitotic**	356	462	2.50E-67
**Infectious disease**	274	348	2.26E-55
**M Phase**	219	268	1.66E-49
**Processing of capped intron-containing pre mRNA**	167	193	9.64E-45
**Metabolism of Proteins**	620	1074	1.16E-41
**mRNA processing (MMU)**	282	398	3.17E-41
**Translation**	135	151	6.03E-40
**Influenza life cycle**	123	136	9.86E-38
**Influenza Infection**	130	147	2.49E-37
**Influenza viral RNA Transcription and Replication**	115	128	1.09E-34
**mRNA Splicing–Major Pathway**	119	134	1.10E-34
**HIV Infection**	173	222	2.63E-34
**mRNA Splicing**	125	144	4.25E-34
**Cap-dependent Translation initiation**	104	114	6.70E-33
**Eukaryotic Translation initiation**	104	114	6.70E-33
**Cellular response to stress**	251	367	8.55E-33
**Mitotic Metaphase and Anaphase**	141	174	1.55E-33
**GTP hydrolysis and joining of the**	98	107	1.99E-31
**SRP-dependent co-translational protein targeting to membrane**	98	107	1.99E-31
**Mitotic Anaphase**	140	173	3.36E-31
**rRNA processing**	144	180	5.05E-31
**L13a-mediated translational silencing of Ceruloplasmin expression**	96	106	7.84E-30
**3’-UTR-mediated translational regulation**	96	106	7.84E-30

### Value of the PDXEx model for predicting tumor response to drugs

**Fig 6:**
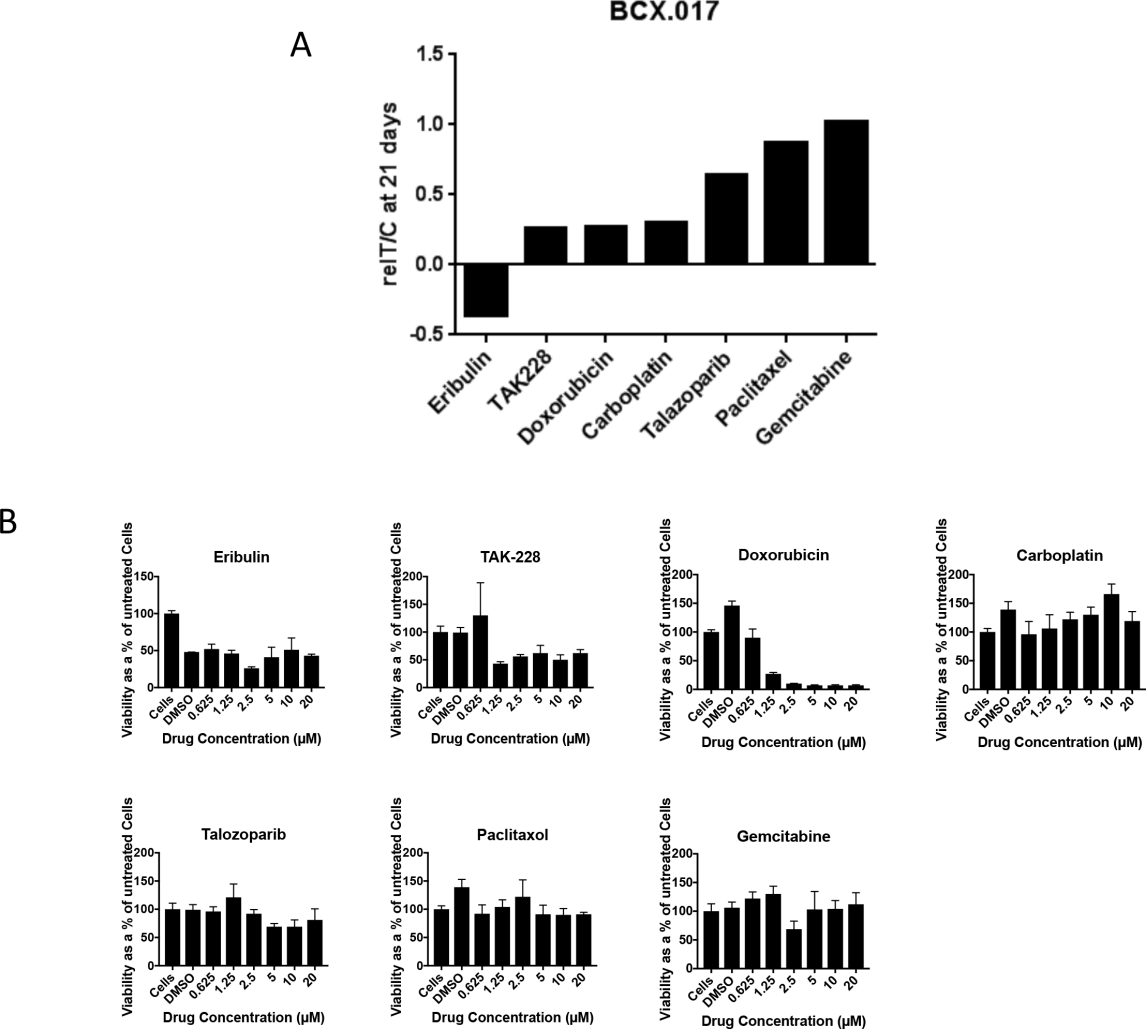
Comparative drug response profile between IBC PDX Bcx017 mouse model and Bcx017 PDXEx tissue model. A) Actively growing (~200–350 mm3) implants in mice were grouped and treated with the indicated drugs for 21days. The graph summarizes data from a previously published in vivo study [69]. B) Tumor cells tagged with a nanoparticle assembly of iron oxide and iron nanoparticles cross-linked with poly-L-lysine (NanoshuttleTM) were bio-printed into ultra low attachment plates. The PDXEx bio-print was treated with the indicated drugs at the indicated dose range for 5 days.

To determine the value of our PDXEx model for predicting tumor response to drugs, we performed a pilot drug screen utilizing this platform and determined the similarity between the drug response profile identified in the *in vitro* screen and the drug response profile identified in a previously described PDX mouse system [53]. Both systems were generated from the same original PDX tumor tissue and treated with the same panel of chemotherapeutic agents: eribulin, TAK228, doxorubicin, carboplatin, talazoparib, paclitaxel, and gemcitabine. The Bcx017 triple-negative IBC PDX model was used in this comparative study. Drug doses ranged from 0.625 μM to 20 μM (Fig 6). The drug response profile identified using our PDXEx model (Fig 6B) mirrored that identified in the corresponding animal study (Fig 5A) except in the case of carboplatin, which showed an anti-tumor effect in the *in vivo* PDX mouse study but not in the PDXEx model. These findings confirmed the value of the PDXEx model in identifying effective tumor-specific therapies. The outcome of this study strengthens our belief that incorporating this PDXEx model within a PDX system to serve as a high-throughput screening platform will permit the identification of effective tumor-specific therapies in a time- and cost-efficient manner.

### Proof-of-principle drug screen utilizing the PDXEx platform

**Fig 7:**
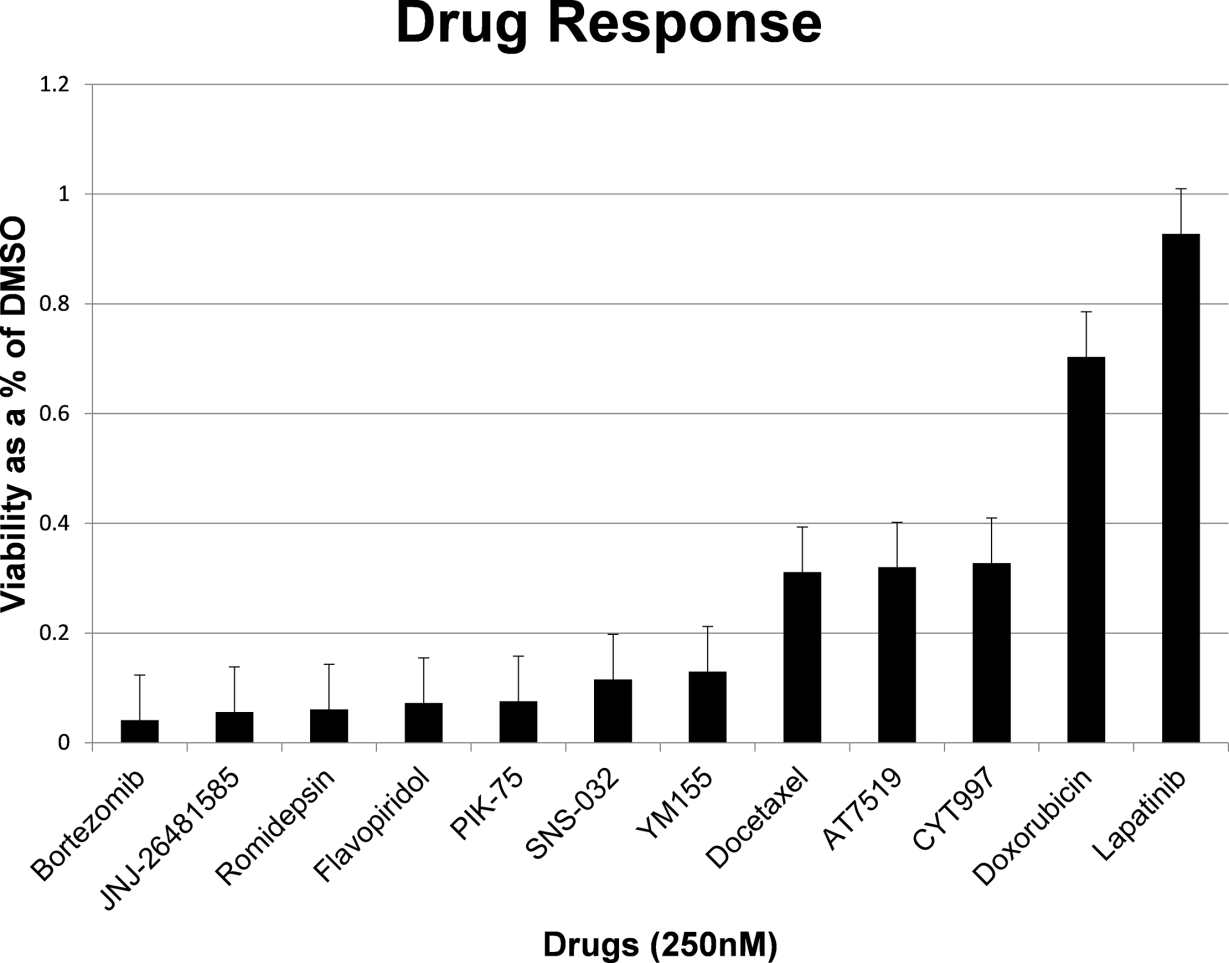
Lead candidates identified from a high throughput drug screen utilizing the PDXEx screening platform. A high throughput small molecule screen was performed utilizing the Bcx087 triple negative PDXEx screening platform and the small molecule Anti-Cancer 386 Compound Library (Selleckchem). The ex vivo tumor tissue array was incubate with the drugs at 37oC for 5 days. The top hits were identified as agents having a superior anticancer effect to that of docetaxel and doxorubicin at 250nM.

Utilizing the Bcx087 bio-printed triple-negative IBC PDXEx platform, we performed a high-throughput small molecule screen with the Anti-Cancer 386 Compound Library (Selleckchem). The drug screen was performed in triplicate, and drug sensitivity as a percentage of the sensitivity of the solvent (DMSO)-treated cells was determined by measuring cellular proliferation using the Cell Titer Glo Luminescent cell viability assay (Promega). The drug screen was performed at drug concentrations of 1 μM, 0.5 μM, and 0.25 μM, and the platform was incubated with the drugs at 37°C for 5 days. Included in this screen were similar concentrations of the standard-of-care drugs doxorubicin and docetaxel [24], which served as positive controls. As the Bcx087 PDX model is a triple-negative IBC model, lapatinib, which targets HER2, was selected as the negative control. Following statistical analysis, we identified the following drugs as our lead candidates with activity superior to that of the standard-of-care drugs at a concentration of 0.25 μM: bortezomib (potent 20s proteasome inhibitor), quisinostat (JNJ-26481585) (second-generation histone deacetylase [HDAC] inhibitor), romidepsin (potent HDAC 1 and 2 inhibitor), flavopiridol (CDK 1, 2, 4, and 6 inhibitor), PIK-75 (p110a inhibitor), SNS-032 (selective inhibitor for CDK2, also inhibits CDK 1, 4, 7, and 9), and YM155 (potent inhibitor of survivin) (Fig 7). In, addition, AT7519 (CDK inhibitor) and CYT997 (microtubule associated) had activity similar to that of the standard-of-care agent docetaxel (Fig 7). Among the 9 top drug candidates, 3 were CDK inhibitors and 2 were HDAC inhibitors. Analyzing the list of genes whose expression levels placed them within the top 25% of the coding genes, we observed that 13 CDK family members were present in this cohort of genes, including CDK1, CDK2, CDK4, and CDK7 and the 9 targets of the CDK inhibitors identified in our screen. Members of the Histone families 1, 2, and 3 were also well represented in this cohort of genes and could have been targets of the identified HDAC inhibitors.

Anticancer drugs developed utilizing 2-dimensional cell culture model systems have had minimal success when translated to the clinic [37]. This failure is often due to the fact that primary tumor cells grown on 2-dimensional monolayer cultures do not accurately mimic cells in the tumor microenvironment [37]. Our group [71] and others [35, 37, 72–73] have identified clinically relevant targets for drug development utilizing 3D cell culture models. However, even 3D cell culture models have limitations as they can still fail to capture the heterogeneity associated with the tumor’s microenvironment. Moreover, often utilized immortalized cancer cell lines, because of their long period of growth outside their natural environment, have acquired unique genetic changes not typically seen in the corresponding tumors in patients [38]. Currently, the most clinically relevant models for drug development are PDX models [38]. Although PDX models are furthering our understanding of cancer biology and our ability to develop more effective cancer therapies, establishing these models is time consuming, cost prohibitive, and resource intensive [38, 48]. In addition, it is not logistically feasible to use PDX model systems for high-throughput screening. One of the approaches to circumvent the need for PDX model systems is the generation of *ex vivo* cultures of primary human tissue. These are preclinical models generated from tumor tissue slices and have been shown to recapitulate the tissue architecture of the original tumor and key features of the tumor microenvironment, including tumor heterogeneity [74]. The use of *ex vivo* model systems in a preclinical setting enables robust quantitative evaluation of clinically relevant anticancer therapies, drug resistance, and biomarkers and thus contributes significantly to identifying successful tumor-specific therapies and improving patients’ quality of life. Another benefit of *ex vivo* models is that by permitting recapitulation of inter-patient heterogeneity, they can be used to differentiate responders from non-responders to a treatment and thus aid in the selection of patients for clinical trials [74]. All previously published organoid models [75–79], including our PDXEx model, are promising models developed for identifying patient-specific therapies. These models have phenotypic and genomic profiles highly similar to those of the original PDX tumor. However, the lack of an immune system and angiogenesis system represents a limitation of the culture system, especially when the aim is to study tumor vasculature and immune interactions. In this regard, we deem it necessary to validate all therapeutic outcomes determined from our PDXEx model system in patient-derived xenografts (PDXs) generated from the same original tumor. PDX models have proved to be useful models for human cancer, because they retain tumor histopathology, including tumor-infiltrating immune cells and generation of tumor vasculature. The importance of validation in PDXs was recently highlighted in a study by Vlachogainnis et al [75]. They tested the ability of their organoid model to recapitulate response to regorafenib, a multiple tyrosine kinase inhibitor blocking oncogenic and angiogenic signaling pathways. No response to regorafenib was observed in their organoid screening assays. However, strong inhibition of tumor growth was observed in an orthotopic human tumor xenograft model. The authors concluded that regorafenib is mainly driven by its antiangiogenic effect.

The IBC PDXEx model that we describe appears to have potential as a solution to the current lack of suitable models for high-throughput identification of effective tumor-specific therapies for IBC. In this study, we clearly showed that i) the tissue architecture of our PDXEx model was very similar to that of the original tumor (Fig 1D), ii) there was a highly significant correlation between the gene expression profiles of the PDXEx model and the original PDX tumor (Fig 3B, 3C and 3D), and iii) our PDXEx model demonstrated a significant predictive power in identifying tumor-specific therapies (Fig 6A and 6B).

Our PDXEx model is simple in design, taking only 11 days to reveal a tissue architecture similar to that of the original PDX tumor (Fig 1). In comparison, it takes about 2 months to grow IBC PDX tumors in mice. At present, we have generated our PDXEx model from PDX tumors and thus have to consider the time taken for the generation of the PDX system. Our future goal is to generate our model directly from human tumor tissue and use it as a screening platform to identify patient-specific therapies. Success in this endeavor will enable us bypass the need for PDX systems and significantly shorten the time it would take to identify patient-specific therapies. Our bio-printed PDXEx screening platform will permit screening of a large number of drugs in just a few days. Incorporating our system as a first-past screening platform to identify the most promising lead agents will cut down on the number of agents that ultimately need to be tested in mice. Our PDXEx screening platform closely replicates the cellular milieu of a tumor microenvironment. To determine the degree to which the tumor microenvironment of the PDX tumor was represented in the PDXEx model derived from this PDX tumor, we analyzed our microarray data from three independent PDX/PDXEx sets for the expression profiles of genes representing the stroma and ECM. The stoma comprises fibroblasts, vasculature (endothelial cells), tumor-associated inflammatory cells and immune cells. Cancer-associated fibroblasts (CAF) are the predominant cell type in the stromal component of the tumor environment [80–81] and are identified by a gene signature comprising ã-smooth muscle actin (ã -SMA), fibroblast activating protein (FAP), tenascin-C (TNC), CD36, IL6 and platelet-derived growth factor ã and ß (PDGFR ã / ß). Our microarray data revealed the levels of expression of fibroblast activating protein (FAP), tenascin-C (TNC), CD36, IL6 and platelet-derived growth factor ã and ß (PDGFR ã / ß). The expression levels of CD34 (endothelial cells), CD24 (B-lymphocytes) and CD3E (T-lymphocytes) were also determined. The ECM is a complex network of macromolecules and is built up of a large collection of biochemically distinct components, including proteins, glycoproteins, proteoglycans, and polysaccharides [82–83]. An acceptable ECM gene signature comprises components of the collagen chain, matrix metalloproteinases (MMP2, MMP11 and MMP14) and ECM regulation and modeling molecules such as SPARC, LoxL1, ADAM12, Plod2 and PALUR [82–83]. Comparing the expression levels of this panel of genes, we observed a high degree of correlation of this panel of genes between each PDX and its corresponding PDXEx. The high correlation of gene expression within this panel of genes suggests a high probability that the our PDXEx model was able to generate a tumor microenvironment similar to that of the original PDX tumor.

Reproducing the tumor microenvironment with a high degree of fidelity is a requirement for any cell culture system used for drug development because the tumor microenvironment not only influences the development of cancer but also has a strong influence on the tumor response to radiation and chemotherapy [84]. The high degree of correlation in gene expression profiles between the PDX tumor and PDXEx model for all 3 of the original PDX tumors shows that the methodology used in the generation of the PDXEx model was both robust and reproducible. Furthermore, the fact that all 3 of the PDXEx models had gene expression similar to that in the original PDX tumor clearly indicates the suitability of our PDXEx model for high-throughput drug screens to identify new and effective therapies and for other studies to further our understanding of IBC.

The diagnosis of IBC relies largely on its clinical presentation; despite a characteristic phenotype, the molecular mechanisms underlying IBC are poorly understood [2]. Analysis of the gene expression profile of our IBC PDXEx model (Table 1) and pathway analysis (Fig 5) (Table 2) also failed to demonstrate a key underlying pathway or driver gene or genes responsible for IBC. The lack of targeted molecular mechanism in triple-negative IBC together with the evidence that patients with triple-negative IBC derive limited benefit from standard therapy [85] highlights the importance of a PDXEx screening platform to identify new and effective therapies for IBC. We predict that by identifying potential therapeutic agents, we could work backwards to further our understanding of the molecular mechanisms mediating the onset and progression of IBC and perhaps identify better therapeutic targets for IBC. We performed a 386-drug Anti-Cancer Compound Library screen utilizing our PDXEx platform, and at a concentration of 250 nM, we identified 7 drugs with a stronger growth inhibitory effect than the standard-of-care drugs and 2 drugs with a growth-inhibitory effect similar to that of the positive control docetaxel (Fig 7). The 9 top candidates were 1 proteosome inhibitor, 2 HDAC inhibitors, 3 CDK inhibitors, 1 PI3K inhibitor, 1 potent inhibitor of survivin, and 1 microtubule inhibitor. HDAC inhibitors [86] have shown favorable outcomes in the treatment of IBC. PI3K/AKT signaling has been suggested to be important for the progression of IBC, and thus PI3K inhibitors might produce favorable outcomes in patients with IBC. CDK inhibitors have been shown to be important for the treatment of hormone-responsive breast cancer [87] and, according to our data, might also be important for the treatment of IBC. The fact that 6 of the 9 top drug candidates identified in our study have published evidence suggesting that the agent might produce favorable outcomes in IBC supports the potential of our preclinical model to identify effective tumor-specific therapies. Our findings also suggest that inhibiting survivin might lead to clinical benefits for patients with triple-negative IBC. Survivin, the smallest member of the inhibitor of apoptosis protein family, is overexpressed in cells of almost all cancers but not in most normal tissues in adults. Survivin expression is required for cancer cell survival, and knocking down its expression or inhibiting its function using molecular approaches results in spontaneous apoptosis. Thus, survivin is an attractive and perhaps ideal target for cancer drug discovery [88]. Studies are currently under way to further our understanding of the role of survivin in IBC and the clinical benefits of inhibitors of survivin as single agents or in combination with other potential therapeutic agents for the treatment of IBC and triple-negative IBC. We are currently planning an *in vivo* study of the Bcx087 PDX model to validate the top drug candidates identified in our bio-printed PDXEx platform.

Although our PDXEx model has potential clinical benefits, it also has one minor drawback. Despite many attempts, we were unable to prolong viability of our *ex vivo* tumor tissue beyond 2 weeks. We do not consider this outcome a major impediment to use of our model since most *ex vivo* models derived from slices of tumor maintain cell viability over culture periods of at least 1 week [74]. Similar to what is true for other *ex vivo* models, use of our PDXEx model as a screening platform requires good availability of biopsy tissue, which could be a limitation in patients with IBC. However, the use of PDX tumor tissue harvested from mice will overcome this limitation, affording us the ability to identify agents with a high likelihood of clinical efficacy before clinical trials or even animal studies. This outcome would undoubtedly improve the efficiency of translating preclinical research to clinical practice. Incorporating *ex vivo* culture of IBC tumors into the development pipeline could help us to achieve this outcome, and this approach has been increasingly used to investigate potential prostate cancer therapeutics [74] using tissue pieces. *Ex vivo* models such as organoid models have been generated from specific cells within tumors with characteristics similar to those of the original tumor [79]. The advantage of these systems is that they can be maintained for long periods of time and used to generate tissue biobanks. Generating such models from IBC tumors is limited by the nature of the disease. At present, no unique structure has been identified in IBC that could be used for the generation of organoid structures; thus, our model might be the best for IBC.

## Conclusions

Further work is needed to determine if our preclinical PDXEx platform will serve as a clinically relevant predictor of tumor response to drugs and improve understanding of the biology of IBC. We have shown in the work described here the importance that *ex vivo* cultures can enable quantitative evaluation of multiple drugs. We postulate that if a drug cannot elicit an anti-proliferative or proapoptotic response in a high proportion of patient tumors in an *ex vivo* system, that drug is unlikely to be effective in the clinics. Here, we show the importance of *ex vivo* cultures enabling the evaluation of drug efficacy using a high-throughput strategy. We are confident that our preclinical *ex vivo* tumor tissue array platform will ultimately be confirmed as a clinically relevant predictor of tumor response to drugs and improve understanding of the biology of IBC.
